# LANGUAGE DIFFERENCE AS A PREDICTOR OF OUTPATIENT FOLLOW-UP AMONG MIDLIFE SPANISH-SPEAKING PATIENTS

**DOI:** 10.1093/geroni/igz038.3146

**Published:** 2019-11-08

**Authors:** Daniel Jimenez, Steven Figiel, Jerad Moxley, Pablo Radusky, Dante Durand

**Affiliations:** 1 University of Miami Miller School of Medicine, Miami, Florida, United States; 2 Lakeview Behavioral Health, Norcross, Georgia, United States; 3 Weill Cornell Medicine, New York, New York, United States; 4 Universidad de Buenos Aires Psychology Research Institute, Buenos Aires, Argentina

## Abstract

The purpose of this study is to determine if psychiatrists’ level of Spanish proficiency impacts patient adherence with follow-up mental health care. We hypothesize that increased Spanish proficiency will increase the likelihood of follow up mental health care. Data were collected via retrospective chart review of Spanish-speaking patients at an adult outpatient center. Our final sample included 201 patients with an average age of 46.5 years. Spanish speaking ability of the clinician was categorized using the standard set by the US Census bureau. The dependent variable of presence or absence of follow-up was dichotomized to whether or not the patient came back for a visit within 6 months of initial evaluation. Odds ratios (ORs) were calculated for each independent variable. Results indicate that Spanish proficiency significantly predicted a follow-up visit. Additionally, patients who were married and those who received psychotherapy were more likely to have a repeat visit. The significance of these results lies in that most research up to date has focused on the English proficiency of the patient as a factor for decreased use of mental health services. Although, effective strategies have been designed to reduce the language barrier in mental health care, Spanish-speaking patients still express a strong preference for bilingual providers. These results may allow identifying which patients are at higher risk of not adhering with follow-up visits. This provides a possibility to anticipate and prevent dropouts from treatment and to implement strategies to support patients that experience a greater burden of social and psychiatric disadvantages.

